# Supplementary material for: Observational evidence of accelerating electron holes and their effects on passing ions
Source: Nat Commun. 2023 Nov 10;14:7276. doi: 10.1038/s41467-023-43033-4 (PMC10638271; doi:10.1038/s41467-023-43033-4)
Supplement: Supplementary file 1 — Supplementary Information [file 41467_2023_43033_MOESM1_ESM.pdf]

Supplementary Information

Observational evidence of accelerating electron holes and their accelerating effects on passing ions

Yue Dong[1], Zhigang Yuan[1]*, Shiyong Huang[1], Zuxiang Xue[1], Xiongdong Yu[1], C. J. Pollock[2], R. B. Torbert[3], and J. L. Burch[4]

[1]*School of Electronic Information, Wuhan University, Wuhan, China*

[2]*Denali Scientific, Fairbanks, AK, USA*

[3]*Physics Department, University of New Hampshire, Durham, NH, USA*

[4]*Southwest Research Institute, San Antonio, Texas, USA*

* Correspondence and requests for materials should be addressed to Zhigang Yuan (y_zgang@vip.163.com)

Supplementary Discussion

Supplementary Figure 1 presents the Magnetospheric Multiscale (MMS) measurements over ten-second interval on August 4, 2017, when the MMS were located at about 20 Earth radii from the Earth in the plasma sheet. A total of 123 slow electron holes (EHs) can be clearly identified by all four MMS satellites in the order of MMS4-MMS1-MMS3-MMS2. The separation of the four MMS satellites in the parallel direction are 5.7 (MMS4-MMS1), 6.9 (MMS1-MMS3) and 5.8 km (MMS3-MMS2), respectively. V_{41}, $V_{13}$and V_{32} represent the average velocities of the slow EHs measured by MMS4-MMS1, MMS1-MMS3 and MMS3-MMS2. From 16:54:48 to 16:54:52, the velocities measured by different satellite pairs gradually converge to local minimum (V_{min}) of the ion velocity distribution function (VDF), indicating that the velocities of the EHs are gradually stable, possibly because the ion beam density is gradually increasing. After 16:54:52, the velocity of the EHs stabilize around the V_{min} in the presence of a strong ion beam. So that the velocity of the EHs stabilize when the ion beam is strong, and might be oscillating or difficult to be measured when the ion beam is weak. The Supplementary Figure 1 illustrates the velocity of the EHs oscillation between the ion core and the ion beam and the whole process of EHs from unstable to stable, which well supports our conclusion. Supplementary Figure 2 shows the theoretical estimates of acceleration/deceleration rates[1]. In the example of the EH C (Fig. 2a), the parameters used in the theoretical prediction are T_a = 7.1 keV, T_r = 2.4 keV, n = 0.06 cm^{-3} and φ_c = 230 eV, where T_a and T_r are the temperatures of electrons and repelled species, respectively. n and φ_c denote the electron density and the maximum repelled potential energy of potential electron hole C. Supplementary Fig. 2c shows the theoretical estimates of the acceleration/deceleration rates using experimental data in units of km s^{-2}. For EH C, the theoretically estimated acceleration rate is around 2×10^4 km s^{-2}, and the observed acceleration rate is 1.3×10^4 km s^{-2}. Similar results are obtained for several other electron holes. Considering measurement errors and theoretical simplification, it is acceptable to have such deviations between observations and theoretical predictions. Therefore, the conclusions are reasonable and supported by the methodology.

Supplementary Figures

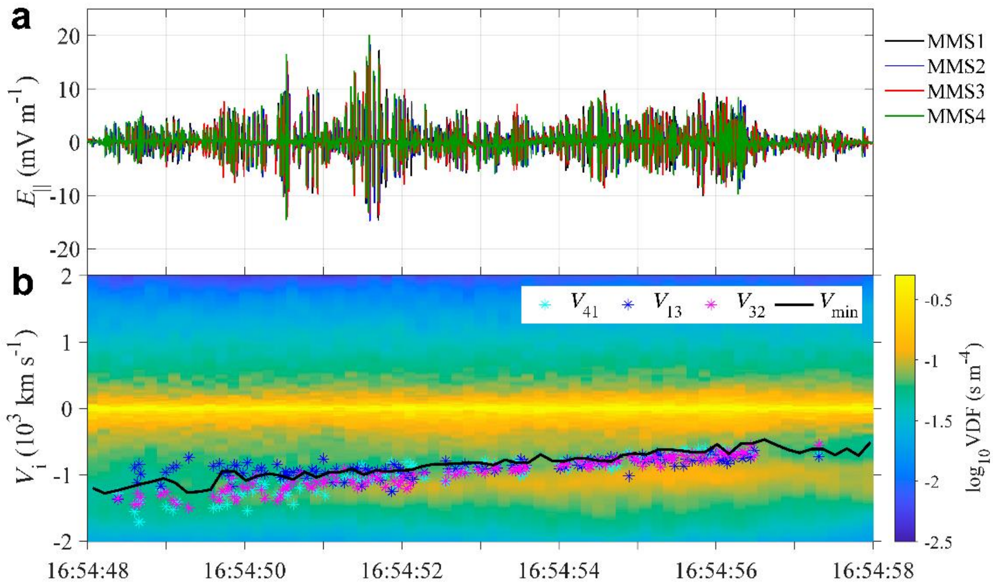

Supplementary Figure 1: Whole process of slow electron holes from unstable to stable state.

a Parallel electric field measured by the four Magnetospheric Multiscale (MMS) spacecraft. **b** Ion one-dimension velocity distribution function (VDF), integrated from the ion three-dimension distribution. The black line represents the local minimum of the ion VDF (V_{min}). The different colored dots indicate the electron hole velocities measured by the different satellite pairs: cyan (V_{41}, MMS4-MMS1), blue (V_{13}, MMS1-MMS3) and magenta (V_{32}, MMS3-MMS2).

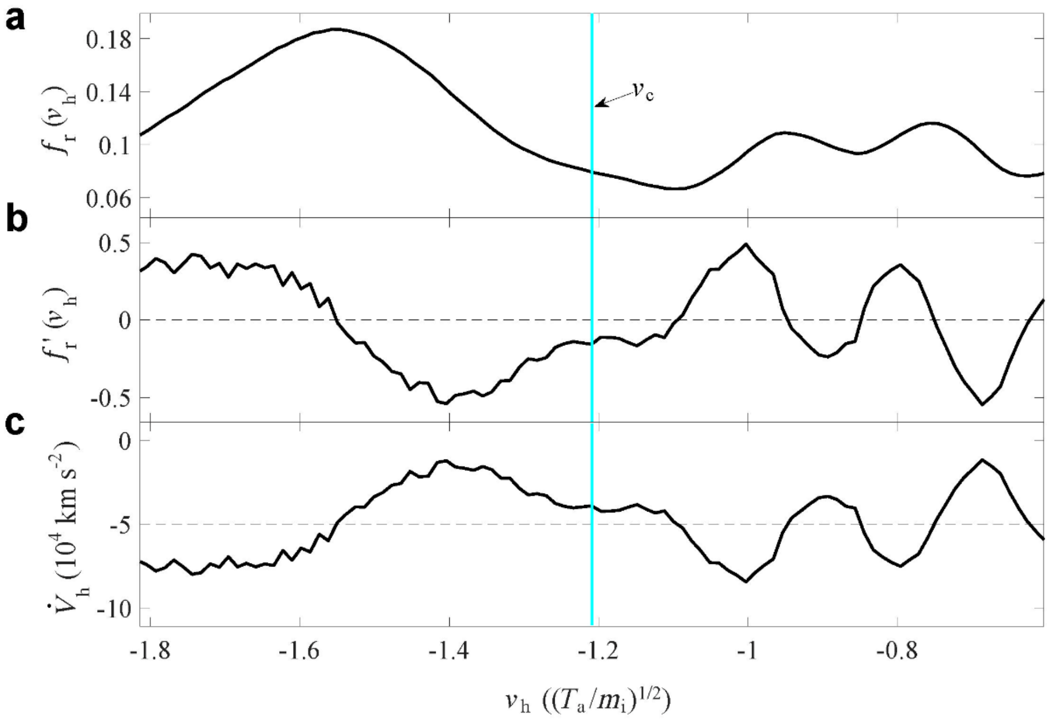

Supplementary Figure 2: Theoretical estimates of acceleration/deceleration rates. **a** The normalized repelled species (ions) distribution function (f_r) vs the normalized electron hole velocity (v_h). **b** The derivative of the normalized repelled species (ions) distribution function ($f_r^{'}$). **c** The electron hole acceleration/deceleration rate in the unit of km s^{-2} obtained from the theoretical prediction (see Supplementary Methods, Theoretical estimates of the acceleration/deceleration rates). The cyan line indicates the observed velocity of the electron hole. The velocity unit in f_r and its derivative is $\sqrt{T_a/m_i}$.

Supplementary Methods

Theoretical estimates of the acceleration/deceleration rates

For electron holes, the attracted species is electrons and repelled species is ions (*e* ↔ *a,* and *i* ↔ *r*). First normalize the ion velocity distribution function (VDF).

$$f_r(v_h) = \frac{f_i(v_h)}{\int f_i(v_h)\mathrm{d}v} \quad \text{(S1)}$$

The velocity unit in $f_r(v_h)$ is $\sqrt{T_a/m_i}$. Eq. (20) in reference Hutchinson, 2023[1] is used to calculate the acceleration/deceleration rates:

$$\dot{v}_h \approx 4\psi^2 f_r^{'}(v_h)/M_a \quad \text{(S2)}$$

Where ψ is the maximum repelled potential energy in units of T_a. $f_r^{'}(v_h)$ is the derivative of $f_r(v_h)$. M_a is the effective mass of the hole, derived from Eq. (21) in reference Hutchinson, 2023[1]:

$$M_a \approx -\frac{16}{3}\psi\sqrt{1+T_a/T_r} \quad \text{(S3)}$$

Then, calculate the nondimensionalized acceleration/deceleration rate $\dot{v}_h$ according to Eq. (S2). Finally, convert the dimensionless $\dot{v}_h$ into units of km s^{-2}, noting that the time unit is $\omega_{pa}^{-1} = \sqrt{\varepsilon_0 m_e/ne^2}$ and the velocity unit is $\sqrt{T_a/m_i}$, so

$$\dot{V}_h = \dot{v}_h \omega_{pa}\sqrt{T_a/m_i} \quad \text{(S4)}$$

Supplementary References

[1] Hutchinson I. H. Ion hole equilibrium and dynamics in one dimension. *Phys. Plasmas* **30**, 032107 (2023).
